# Recombinant attenuated M.tuberculosis-SIVgag(rAMtb-gag) vaccination primes for SIV-specific CD8 T cell response that are boosted by Ad5-SIVgag in mice

**DOI:** 10.1186/1742-4690-9-S2-P263

**Published:** 2012-09-13

**Authors:** U Kadayam Ranganathan, M Larsen, K Abel, WR Jacobs, G Fennelly

**Affiliations:** 1 Albert Einstein College of Medicine, Bronx, NY, USA; 2 Dept of Microbiology and Immunology, UNC School of Medicine, Chapel Hill, NC, USA; 3 Howard Hughes Medical Institute, Albert Einstein College of Medicine, USA

## Background

BCG vaccination is no longer advised in children with HIV infection due to the risk of disseminated disease. As a safer alternative to BCG and as a vector for HIV vaccination, we developed a novel vaccine based on an M. tb mutant that contains genetic deletions in essential nutrients (pantothenate and Leucine) and immune modulating pathways (ΔSecA2).

## Methods

To optimize T cell responses against SIV after vaccine priming with a candidate ΔPanCD ΔLeuCD ΔSecA2 M. tuberculosis (mc^2^6208) strain expressing the codon optimized SIVmac239 gag (designated mc^2^6435), we boosted with Adenovirus 5 expressing SIV gag in 5-6 week old C57/BL6 mice. Lymphocytes from blood, lung and spleen cells were collected 2 and 6 weeks after boosting to detect SIV gag-specific CD8 T cells by tetrameric staining for the H-2Db haplotype AL11 tetramer (AAVKNWMTQTL) and flow cytometric analysis.

## Results

To determine the relative priming ability of rAMtb, rBCG, DNA vaccine, and rAd expressing the relevant Gag sequence, several groups of mice (5 per group) were immunized according to various prime/boost schedules. In general, Ad5gag boosting enhanced T cell responses after either mc^2^6435 or BCG-SIV priming compare to mycobacterial priming alone.

## Conclusion

mc^2^6435, mc^2^6206 (ΔPanCD ΔLeuCD) expressing SIV gag and rBCG boosted with Ad5gag had comparable response in the lung at 2 wks (2-6%). Although the most frequent SIV-specific CD8+ T cell responses were observed after Ad5gag boosting among splenocytes (7%) in BCG primed mice, significant responses (3-6% of all CD8+ T cells) were observed after Ad5gag boosting in mice primed with either rAMtb-gag strain mc^2^6435 or mc^2^6206. These results suggest that SIV-specific T cell responses after rAMtb-gag priming can be boosted by the Ad5gag. rAMtb-HIV is a safe candidate vaccine for infants that is likely to prime for T cell responses against both HIV and TB.

